# Prevalence, morphology, and molecular characterization of trypanosomes in bulbuls from Thailand

**DOI:** 10.1051/parasite/2026036

**Published:** 2026-07-08

**Authors:** Pornchai Pornpanom, Carolina Romeiro Fernandes Chagas, Nikom Srikacha, Surya Paudel, Arif Ciloglu, Kannawee Swangneat, Morakot Kaewthamasorn

**Affiliations:** 1 Akkhraratchakumari Veterinary College, Walailak University Nakhon Si Thammarat 80160 Thailand; 2 Informatics Innovation Center of Excellence, Walailak University Nakhon Si Thammarat 80160 Thailand; 3 One Health Research Center, Walailak University Nakhon Si Thammarat 80160 Thailand; 4 State Scientific Research Institute Nature Research Centre Vilnius 08412 Lithuania; 5 Department of Animal Science, Faculty of Natural Resources, Rajamangala University of Technology Isan Sakon Nakhon 47160 Thailand; 6 Department of Infectious Diseases and Public Health, Jockey Club College of Veterinary Medicine and Life Sciences, City University of Hong Kong Hong Kong Special Administrative Region PR China; 7 Jockey Club College of Veterinary Medicine and Life Sciences Research Centre for Applied One Health Research and Policy Advice, City University of Hong Kong Hong Kong Special Administrative Region PR China; 8 Department of Parasitology, Faculty of Veterinary Medicine, Erciyes University Kayseri 38280 Türkiye; 9 Department of Pathology, Faculty of Veterinary Science, Chulalongkorn University Bangkok 10330 Thailand; 10 Center of Excellence in Veterinary Parasitology, Department of Pathology, Faculty of Veterinary Science, Chulalongkorn University Bangkok 10330 Thailand

**Keywords:** Avian trypanosomes, Bulbuls, Protozoan parasite, *Pycnonotus*, *SSU rRNA*

## Abstract

Birds represent the second most species-rich class of vertebrates, comprising more than 10,000 extant species worldwide, including wild and domesticated forms, as well as migratory and resident species. Bulbuls (Family Pycnonotidae) are important seed dispersers, and keeping them in cages is a long-standing tradition in Thai culture. Health assessments of these birds are therefore essential for sustaining cage-bird keeping, supporting local livelihoods, while also supporting biodiversity conservation. This study aimed to investigate the prevalence, morphology, and molecular characteristics of trypanosomes in Olive-winged bulbuls (*Pycnonotus plumosus*, OWB), Streak-eared bulbuls (*Pycnonotus conradi*, SEB), and Yellow-vent bulbuls (*Pycnonotus goiavier*, YVB) captured in Thailand. Blood samples were collected from 121 bulbuls (26 OWBs, 79 SEBs, and 16 YVBs). Three Giemsa-stained blood smears were prepared per sample, and genomic DNA was extracted. Additionally, nested-PCR and sequence analysis of the small subunit ribosomal ribonucleic acid gene (*SSU rRNA*) were performed. As a result, microscopic examination revealed *Trypanosoma* spp. infections in seven SEBs and ten YVBs, and up to nine putative morphotypes of *Trypanosoma* spp. were identified. Nested-PCR revealed a higher prevalence (11.39% in SEBs and 75.00% in YVBs) compared to microscopic examination (8.86% in SEBs and 62.50% in YVBs). Sequence analysis of all 21 nested-PCR positive samples further revealed multiple trypanosome strain/species infections. Collectively, these findings provide baseline data to support veterinary diagnostic laboratories and inform future epidemiologic and molecular investigations of avian trypanosomes in Thailand.

## Abstract (fr)

Les oiseaux constituent la deuxième classe de vertébrés la plus diversifiée, avec plus de 10 000 espèces actuelles à travers le monde, incluant des formes sauvages et domestiques, ainsi que des espèces migratrices et sédentaires. Les bulbuls (famille des Pycnonotidae) jouent un rôle important dans la dispersion des graines, et leur élevage en cage est une tradition ancestrale en Thaïlande. Le suivi sanitaire de ces oiseaux est donc essentiel pour pérenniser cet élevage, soutenir les moyens de subsistance locaux et contribuer à la conservation de la biodiversité. Cette étude visait à examiner la prévalence, ainsi que les caractéristiques morphologiques et moléculaires des trypanosomes chez le bulbul aux ailes olive (*Pycnonotus plumosus*, BAO), le bulbul de Conrad (*Pycnonotus conradi*, BC) et le bulbul goyavier (*Pycnonotus goiavier*, BG) capturés en Thaïlande. Des échantillons de sang ont été prélevés sur 121 bulbuls (26 BAO, 79 BC et 16 BG). Trois frottis sanguins colorés au Giemsa ont été préparés pour chaque échantillon, et l'ADN génomique a été extrait. De plus, une PCR nichée et un séquençage du gène de l'ARN ribosomique de la petite sous-unité (ARNr 18S) ont été réalisés. L'examen microscopique a révélé des infections à *Trypanosoma* spp. chez sept BC et dix BG, et jusqu'à neuf morphotypes putatifs de *Trypanosoma* spp. ont été identifiés. La PCR nichée a révélé une prévalence plus élevée (11,39 % chez BC et 75,00 % chez BG) que l'examen microscopique (8,86 % chez BC et 62,50 % chez BG). Le séquençage des 21 échantillons positifs à la PCR nichée a par ailleurs révélé des infections par plusieurs souches/espèces de trypanosomes. Collectivement, ces résultats fournissent des données de référence pour soutenir les laboratoires de diagnostic vétérinaire et éclairer les futures enquêtes épidémiologiques et moléculaires sur les trypanosomes aviaires en Thaïlande.

## Introduction

Birds (Class Aves) represent the one of most species-rich vertebrates, comprising more than 10,000 extant species worldwide [10, 15], including both wild and domesticated forms. Among them, migratory species undertake regular intercontinental movements along well-defined flyways, crossing diverse ecological regions throughout their annual cycles. Such long-distance movements increase exposure to a wide range of parasite communities [13, 31] and hematophagous arthropod vectors, thereby facilitating pathogen exchange across geographic boundaries and potentially contributing to the dissemination of infectious agents among migratory, resident, and domestic bird populations [18].

The East Asian-Australasian flyway is one of the major migratory routes, supporting nearly 400 migratory bird species [44, 64]. Thailand serves as a key stopover and wintering site along this flyway [21]. In this country, avian trypanosomes (Euglenozoa, Kinetoplastea, Trypanosomatidae), vector-borne hemoflagellates infecting birds globally [17], are found in wild birds [35, 40] and domestic chickens [9, 36]. This suggests that the local environmental conditions may be favorable for avian trypanosome transmission, making this country a potential hotspot for parasite transmission among domestic birds, resident wild birds, and migratory birds. However, available information about the diversity, epidemiology, and transmission dynamics is limited. This knowledge gap highlights the need for investigations to clarify our understanding of avian trypanosomes and establish baseline data supporting health monitoring, bird conservation, and future research.

Avian trypanosomes are transmitted by hematophagous arthropods, including biting midges, black flies, hippoboscids, mosquitoes, and sandflies [7, 34, 41, 48, 49, 56, 65]. Thailand harbors substantial diversity of these vectors, with more than 100 recorded species of black flies and biting midges [55, 56]. Species such as *Culicoides huffi*, the *Simulium asakoae* complex, *Simulium chumpornense*, and *Simulium khelangense* have been proposed as potential vectors of avian trypanosomes in the country [46, 47, 56]. Transmission may occur during vector prediuresis [59], whereby infective stages are excreted in urine droplets that contaminate abraded skin or conjunctival surfaces [3, 19, 59]. Ingestion of infected vectors has also been suggested as an alternative transmission route [47].

Although more than 100 species of avian trypanosomes have been described, many were insufficiently illustrated or described and lack information on host and vector specificity [58, 59]. In bulbuls (family Pycnonotidae), only three species have been documented: *Trypanosoma avium* [4, 14], *Trypanosoma brimonti* [28], and *Trypanosoma pycnonoti* [25], with a notable absence of genetic characterization. Although *Trypanosoma* species have traditionally believed to exhibit strict host specificity, this assumption has not been rigorously validated [58]. Notably, *T. avium* has been reported in a wide range of passerine species [4], owls [35], as well as domestic chickens (family Phasianidae) [36], supporting the view that this parasite exhibits low host specificity [43].

In Nakhon Si Thammarat Province, seven bulbul species have been documented [16], representing a subset of the 41 species recorded nationwide [8]. Bulbuls play important ecological roles as seed dispersers [51] and are legally protected for most species [29]. However, certain species, particularly the Red-whiskered bulbul (*Pycnonotus jocosus*, RWB), are commercially valuable and frequently targeted for the bird trade [54]. Cage-keeping of bulbuls, including RWB and their hybrids, constitutes an integral component of Thai cultural practices, although only RWBs are legally permitted for captive breeding [52]. Accordingly, health assessments of bulbuls are relevant not only to wildlife conservation but also to sustainable cage-bird management and local livelihoods.

Thus, this study aimed to investigate the prevalence and detailed characterization of trypanosomes in Olive-winged bulbuls (*Pycnonotus plumosus*, OWB), Streak-eared bulbuls (*Pycnonotus conradi*, SEB), and Yellow-vent bulbuls (*Pycnonotus goiavier*, YVB), using an integrative approach combining microscopic examination and nested-PCR targeting the small subunit ribosomal RNA (*SSU rRNA*) gene [36, 58]. Additionally, sex-biased infection had been reported in other parasites, such as *Plasmodium* [11]. Thus, this study also investigated sex-biased trypanosome infection. As part of an ongoing project on avian malaria and other blood parasites in wild passerine birds in southern Thailand, the data generated from bulbuls captured in green spaces at Walailak University, Nakhon Si Thammarat, may contribute to routine health assessments in wildlife and exotic pet care, supporting local livelihoods and promoting bird conservation.

## Materials and methods

### Ethical considerations

All sampling procedures in animals were reviewed and approved by the Walailak University Institutional Animal Care and Use Committee (Approval number: WU-ACUC-67028). Handling of samples and molecular analysis of blood parasites were conducted in compliance with the regulations of the Institutional Biosafety Committee (IBC) of Walailak University (Approval number: WU-IBC-67-028).

### Birds capture and blood collection

During the rainy season in Southern Thailand (August to September 2024), a total of 121 bulbuls (26 OWBs, 79 SEBs, and 16 YVBs) were captured using mist nets in green spaces (grassland, shrubland, and wetland) of Walailak University, Nakhon Si Thammarat, Thailand (8° 38′ N, 99° 53′ E). The sampled birds included 19 female and seven male OWBs, 47 female and 32 male SEBs, and seven female and nine male YVBs, with sex determined by PCR [20]. The captured birds were transported to the Laboratory of Veterinary Clinical Pathology, Akkhraratchakumari Veterinary College, for ringing and blood collection. A small amount of blood (~100 μL) was collected from each bird using the capillary tube method [27, 33]. Immediately after collection, three fresh blood smears were prepared and air-dried using an electrical fan. The remaining blood was stored in ethylenediaminetetraacetic acid (EDTA) tubes (Quetainer^TM^, Cangzhou Fukang Medical Supplies, Hebel, PR China) and refrigerated for subsequent molecular analyses. The dried blood smears were fixed in absolute methanol and stained with 10% Giemsa solution for one hour [57]. Following blood collection, the birds were released into the wild.

### Microscopic, morphologic, and morphometric analysis

Giemsa-stained blood smears were examined for the presence of trypanosomes under an Olympus BX43 light microscope (Olympus, Tokyo, Japan), equipped with a DP27 digital camera (Olympus) and operated using CellSens imaging software (version 1.18, Olympus). Blood smears were initially screened at 400× magnification across the entire smear and then re-evaluated at 1000× magnification (for 100 fields) to confirm trypanosome infection [36]. Detected trypomastigotes were photographed using an oil-immersion lens (1000×) for morphological analysis and compared with previously reported descriptions [2, 4–7, 14, 25, 28, 32, 36, 42, 45, 58, 60, 63]. Additionally, parasite intensity in positive samples was estimated by counting the number of trypomastigotes observed in 10 microscopic fields at 100x (10x objective lens) magnification [40].

### Nested-PCR for the SSU rRNA gene of avian trypanosomes

Genomic DNA was extracted using a Blood Genomic DNA Extraction Mini Kit (FavorPrep, Pingtung, Taiwan), following the manufacturer’s instructions and subsequently used as templates to amplify the small subunit ribosomal ribonucleic acid (*SSU rRNA*) gene, as described in a previous report [58]. Nested-PCR was performed with an initial denaturation at 95 °C for 5 minutes, followed by 35 cycles of 95 °C for 1 minute, 45 °C (primary reaction) or 58 °C (secondary reaction) for 30 s, and 72 °C for 1 minute, with a final extension at 72 °C for 10 min.

PCR reactions were performed in a total volume of 20 μL, containing 10 μL of PCR Master Mix (OmniPCR™, Bio-Helix, New Taipei City, Taiwan), 1 μL of each primer (external primers: Tryp763 (5′–CAT ATG CTT GTT TCA AGG AC–3′) and Tryp 1016 (5′–CCC CAT AAT CTC CAA TGG AC–3′); internal primers: Tryp99 (5′–TCA ATC AGA CGT AAT CTG CC–3′) and Tryp957 (5′–CTG CTC CTT TGT TAT CCC AT–3′); 10 μM), 6 μL of ultrapure water, and 2 μL of DNA template (concentration ranging from 0.2 to 121 ng/μL). A non-template control and a positive control [*Trypanosoma* KU127 (GenBank accession no: MH549542] were included in each PCR run. After electrophoresis on a 1.5% agarose gel containing RedSafe™ nucleic acid stain (iNtRON Biotechnology, South Korea), PCR amplicons (~770 bp) of the *SSU rRNA* gene were purified and sent to Macrogen (Seoul, South Korea) for Sanger sequencing.

### Sequence analysis and phylogeny

All sequences from nested-PCR positive samples were examined to determine whether they were single or mixed infections. Clean, single-peak electropherograms was classified as single infections, whereas sequences with double peaks, stutter peaks, or dye blob [1] were considered mixed infections. All sequences were compared with reference trypanosome sequences in the National Center for Biotechnology Information (NCBI) database using the Basic Local Alignment Search Tool (BLAST) to confirm genus-level identity. For mixed infections, sequences with high-quality chromatograms, minimal double peaks, and unambiguous bases were manually trimmed at both ends to obtain the Tryp99-Tryp957 fragment of the *SSU rRNA* gene using BioEdit [22]. The trimmed sequences were considered the dominant genotype among the multiple genotypes detected in the sample, based on the assumptions from the previous report [38]. These sequences were subsequently used for phylogenetic analysis.

The phylogenetic analysis was constructed using three haplotypes (isolated from mixed-infection samples) obtained from this study, together with 54 *SSU rRNA* gene sequences of avian trypanosomes retrieved from GenBank selected based on previously published studies [7, 24, 35, 36, 41, 45, 58, 62, 65]. Sequences of two amphibian trypanosomes (*Trypanosoma rotatorium* (AJ009161) and *Trypanosoma mega* (AJ009157)) were included as outgroups. The consensus length (909 bp, including gaps) was used to generate a Maximum-likelihood phylogenetic tree with 1,000 bootstrap replicates, implemented in MEGA 11 [50]. The best-fit substitution model was the Kimura 2-parameter with gamma distribution and invariant sites (K2 + G + I). Genetic divergence between sequences was estimated using the Jukes–Cantor model [23], assuming equal substitution rates for all nucleotide changes. Obtained sequences were deposited in GenBank (Accession number: PX992725–PX992727).

### Statistical analysis

The prevalence of trypanosomes was estimated based on the results of both microscopic and molecular analyses. The 95% confidence intervals (CIs) were calculated using the “*binom.approx*” function from the “*epitools*” package in R version 4.5.3 [39]. The chi-square test (χ^2^) and Fisher’s exact test were applied to assess differences in the prevalence of blood parasite infections (based on nested-PCR results) between sexes (male and female bulbuls) and species of bulbuls (OWBs, SEBs, and YVBs). Statistical significance was set at *p* < 0.05. To assess differences in morphometric parameters among trypanosome morphotypes detected in SEBs, the Kruskal–Wallis test was performed, followed by Bonferroni-corrected multiple comparisons. A *p-*value of <0.05 was considered statistically significant. All statistical analyses were performed using R software version 4.5.3 [39].

## Results

### Prevalence of trypanosomes infection

Microscopic and molecular analyses revealed no trypanosome infections in OWBs, while microscopic examination detected trypanosome infections in seven SEBs (8.86%, 95% CI: 2.59%–15.13%) and ten YVBs (62.50%, 95% CI: 38.78%–86.22%). Parasitemia levels were low, with an average of <1 trypomastigotes observed per LPF. *Haemoproteus* sp. were also detected; however, these findings were fragmentary and were not included in this study. Through nested-PCR, trypanosome infections were detected in nine SEBs (11.39%, 95% CI: 4.39%–18.40%) and twelve YVBs (75.00%, 95% CI: 53.78%–96.22%). Among microscopically positive SEBs, two were negative by nested PCR. Additionally, the statistical test revealed that prevalence of *Trypanosoma* sp. in YVBs was significantly higher than that of SEBs and OWBs, χ^2^ = 44.49, *p* < 0.001 (Table 1). In contrast, Fisher’s exact test revealed no significant difference in prevalence between male SEBs (15.63%, 95% CI: 3.04%–28.21%) and female SEBs (8.51%, 95% CI: 0.53%–16.49%). Similarly, no significant difference in prevalence was found between male YVBs (77.78%, 95% CI: 50.62%–100%) and female YVBs (71.43%, 95% CI: 37.96%–100%).

**Table 1:** Molecular prevalence of trypanosomes in Olive-winged bulbuls (*Pycnonotus plumosus*, OWBs), Streak-eared bulbuls (*Pycnonotus conradi*, SEBs) and Yellow-vent bulbuls (*Pycnonotus goiavier*, YVBs) from Thailand.

Host	Sex	Positive samples	Negative samples	Prevalence (95% CI)	χ^2^ (*p*-value)/Fisher’s exact	
All birds	Male (*n* = 48)	12	36	25.00% (12.75%–37.25%)	χ^2^ = 2.42^b^	
	Female (*n* = 73)	9^a^	64	12.33% (4.79%–19.87%)	df = 1	
	Total (*n* = 121)	21	100	17.36% (10.61%–24.10%)	(*p* = 0.1199)	
OWBs	Male (*n* = 7)	0	7	0%	N/A	χ^2^ = 44.49^c^ df = 2 (*p* < 0.001)
	Female (*n* = 19)	0	19	0%		
	Total (*n* = 26)	0	26	0%		
SEBs	Male (*n* = 32)	5	27	15.63% (3.04%–28.21%)	Odds ratio = 1.97^d^ (*p* = 0.4732)	
	Female (*n* = 47)	4^a^	43	8.51% (0.53%–16.49%)		
	Total (*n* = 79)	9	70	11.39% (4.39%–18.40%)		
YVBs	Male (*n* = 9)	7	2	77.78% (50.62% – 100%)	Odds ratio = 1.37^d^ (*p* = 1)	
	Female (*n* = 7)	5	2	71.43% (37.96% – 100%)		
	Total (*n* = 16)	12	4	75.00% (53.78% – 96.22%)		

^a^Two female SEBs were negative for nested-PCR but trypomastigotes of *Trypanosoma* sp. were found in Giemsa-stained blood smears.

^b^Chi-square test was used to determine the difference in prevalence between male and female bulbuls.

^c^Chi-square test was used to determine the difference in prevalence between bulbul species.

^d^Fisher’s exact was used to determine the difference in prevalence between male and female bulbuls.

### Morphological characteristics of trypanosomes in streak-eared bulbuls

**Figure 1:**
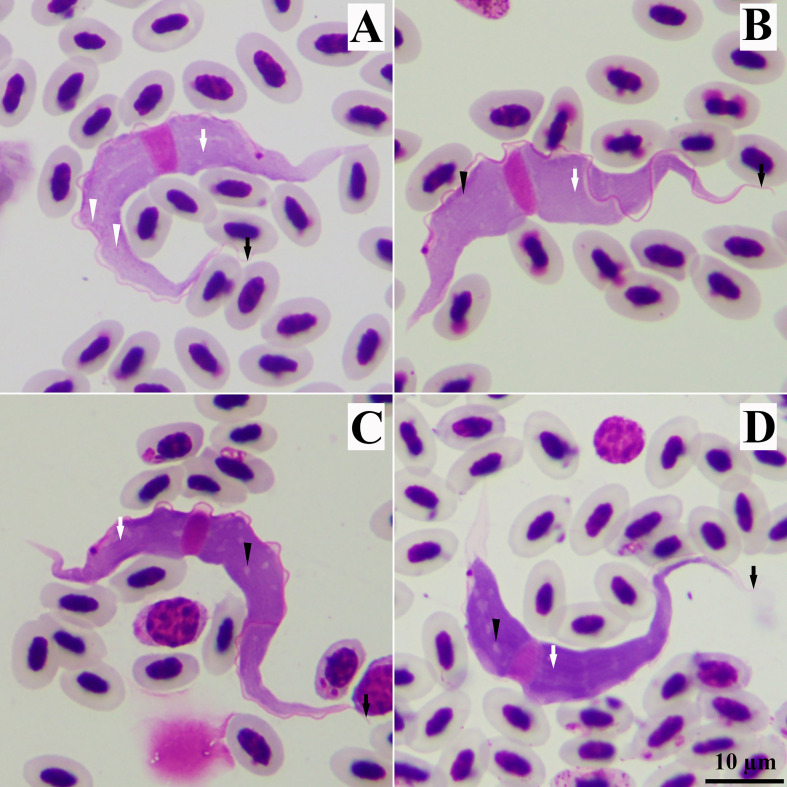
Large trypomastigote of *Trypanosoma* sp. found in Streak-eared bulbuls (*Pycnonotus conradi*). Morphotype SEBL-I showed large trypomastigotes with purple cytoplasm and distinct scattered azurophilic granules (A), morphotype SEBL-II showed large trypomastigotes with purple cytoplasm without distinct scattered azurophilic granules (B), morphotype SEBL-III showed large trypomastigotes with deep purple cytoplasm and scattered pale spots (C), and morphotype SEBL-IV showed large trypomastigotes with deep purple cytoplasm, scattered pale spots and pale/colorless at both ends (D). Note: Free flagellum = black arrow, longitudinal striations (myonemes) = white arrow, pale spots = black arrow head, azurophilic granules = white arrow head.

**Figure 2:**
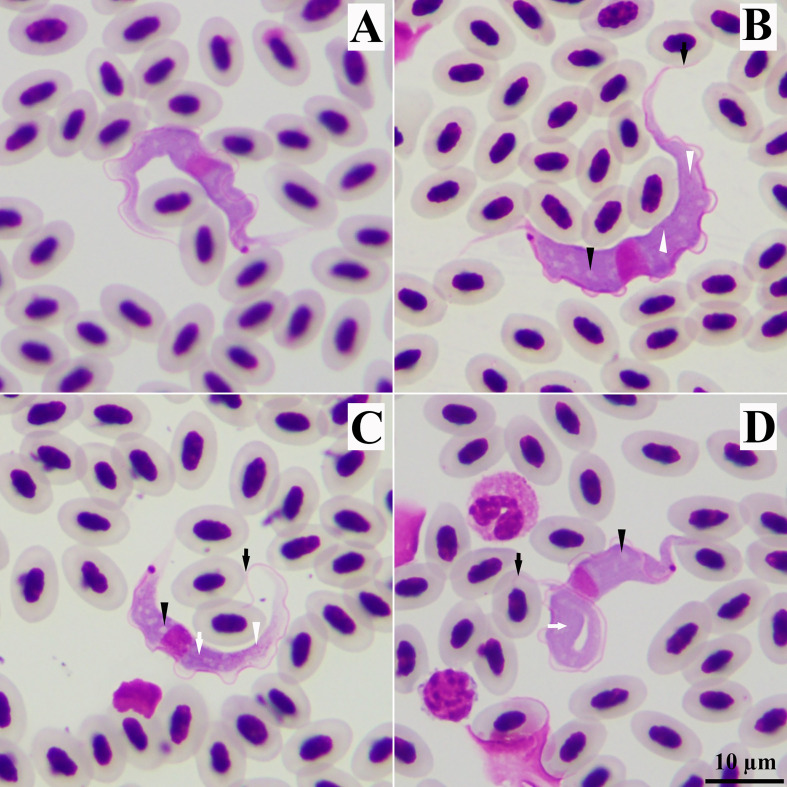
Small trypomastigote of *Trypanosoma* sp. found in Streak-eared bulbuls (*Pycnonotus conradi*), morphotypes SEBS. Note: Free flagellum = black arrow, longitudinal striations (myonemes) = white arrow, pale spots = black arrow head, azurophilic granules = white arrow head.

Trypanosomes found in SEBs showed five morphotypes (Table 2), including four large morphotypes (SEBL-I, SEBL-II, SEBL-III, and SEBL-IV, Figs. 1A–1D) and one small morphotype (SEBS, Figs. 2A–2D). Trypomastigotes of morphotype SEBL-I showed longitudinal striations (myonemes). The cytoplasm was purple and contained scattered azurophilic granules (Fig. 1A). The kinetoplast was small, round to oval, and stained reddish-purple. The undulating membrane was distinct and generally shallow. The oval, pinkish-purple nucleus was located near the center of the trypomastigote. The free flagellum was 5.51 ± 1.08 μm in length (Table 3). For morphotype SEBL-II, the general characteristic features were similar to those of morphotype SEBL-I, but lacked distinct scattered azurophilic granules (Fig. 1B).

**Table 2:** Microscopic examination of trypanosomes in Streak-eared bulbuls (SEBs) and Yellow-vent bulbuls (YVBs) from Thailand.

Birds	Morphotypes of trypanosomes*								
	SEBL-I	SEBL-II	SEBL-II	SEBL-IIV	SEBS	YVBL-I	YVBL-II	YVBL-III	YVBS
SEBs									
WU27	Positive	Negative	Positive	Negative	Positive	–	–	–	–
WU36	Positive	Positive	Negative	Negative	Positive	–	–	–	–
WU58	Negative	Positive	Negative	Positive	Positive	–	–	–	–
WU65	Negative	Positive	Negative	Negative	Positive	–	–	–	–
WU71	Positive	Positive	Negative	Negative	Positive	–	–	–	–
WU179	Positive	Negative	Positive	Negative	Positive	–	–	–	–
WU186	Negative	Positive	Negative	Positive	Negative	–	–	–	–
YVBs									
WU01	–	–	–	–	–	Negative	Negative	Negative	Positive
WU28	–	–	–	–	–	Negative	Positive	Negative	Positive
WU33	–	–	–	–	–	Negative	Positive	Negative	Positive
WU40	–	–	–	–	–	Positive	Positive	Negative	Positive
WU47	–	–	–	–	–	Negative	Negative	Negative	Positive
WU105	–	–	–	–	–	Negative	Negative	Positive	Negative
WU107	–	–	–	–	–	Negative	Positive	Negative	Negative
WU112	–	–	–	–	–	Negative	Positive	Positive	Negative
WU184	–	–	–	–	–	Negative	Negative	Negative	Positive
WU185	–	–	–	–	–	Negative	Negative	Negative	Positive

*Morphotypes of trypanosomes found in Steak-eared Bulbuls, including SEBL-I = Large trypomastigote with purple cytoplasm and distinct scattered azurophilic granules; SEBL-II = Large trypomastigote with purple cytoplasm without distinct azurophilic granules; SEBL-III = Large trypomastigote with deep purple cytoplasm and scattered pale spots; SEBL-IV = Large trypomastigote with deep purple cytoplasm, distinct scattered pale spots and pale/colorless at both ends, and SEBS = Small trypomastigote with well-pronounced undulating membrane.

** Morphotypes of trypanosomes found in Yellow-vent Bulbuls, including YVBL-I = Large trypomastigote with pale purple cytoplasm and scattered azurophilic granules; YVBL-II = Large trypomastigote with purple cytoplasm with scattered pale spots; YVBL-III = Large trypomastigote with deep purple cytoplasm with scattered pale spots; and YVBS = Small trypomastigote with well pronounced undulating membrane.

**Table 3:** Morphometry of five morphotypes of *Trypanosoma* sp. found in Streak-eared bulbuls (*Pycnonotus conradi*) from Thailand.

Features^*^	Unit	Measurements**			
		SEBL-I (*n* = 9)	SEBL-II (*n* = 12)	SEBL-III (*n* = 4)
AK	μm^2^	1.00 ± 0.28 (0.53–1.30)	1.06 ± 0.25 (0.60 – 1.36)	0.85 ± 0.16 (0.65 – 1.02)
AN	μm^2^	25.42 ± 3.15 (20.07–30.83)^a^	23.38 ± 2.26 (19.79 – 27.18)^ab^	23.01 ± 2.50 (19.68 – 25.42)^ab^
AT	μm^2^	271.75 ± 46.17 (229.61–369.58)^a^	272.21 ± 32.24 (230.93 – 335.12)^a^	244.37 ± 19.52 (219.71 – 266.44)^ab^
BW	μm	7.96 ± 1.26 (5.83–9.76)^a^	7.58 ± 0.70 (6.11 – 8.60)^a^	6.71 ± 0.42 (6.27 – 7.29)^ab^
FF	μm	5.51 ± 1.08 (3.31–7.20)	4.57 ± 1.60 (2.41 – 7.55)	3.69 ± 0.74 (2.85 – 4.63)
KN	μm	14.86 ± 1.64 (12.87–18.06)	14.62 ± 1.35 (12.63 – 16.59)	14.40 ± 0.92 (13.42 – 15.64)
NA	μm	34.66 ± 3.45 (30.24–41.14)	36.11 ± 2.37 (31.83 – 39.21)	36.75 ± 3.32 (34.27 – 41.59)
PA	μm	62.90 ± 6.22 (54.04–70.67)	64.45 ± 4.52 (58.89 – 72.08)	66.42 ± 2.47 (64.01 – 69.35)
PK	μm	14.52 ± 2.15 (11.08–17.32)	14.93 ± 2.32 (11.90 – 18.61)	15.01 ± 1.18 (13.52 – 16.37)
PN	μm	27.99 ± 2.70 (26.62–32.05)	28.84 ± 2.64 (25.66 – 32.32.74)	29.56 ± 1.22 (27.86 – 30.73)
AN/AT	μm	0.09 ± 0.01 (0.07–0.11)	0.09 ± 0.01 (0.07 – 0.10)	0.10 ± 0.02 (0.07 – 0.12)
BW/PA	μm	0.13 ± 0.02 (0.10–0.18)	0.12 ± 0.02 (0.08 – 0.14)	0.10 ± 0.01 (0.09 – 0.11)
PK/PA	μm	0.23 ± 0.03 (0.18–0.26)	0.23 ± 0.03 (0.19 – 0.27)	0.23 ± 0.03 (0.19 – 0.26)
PN/KN	μm	1.89 ± 0.09 (1.76–1.99)	1.98 ± 0.19 (1.68 – 2.23)	2.06 ± 0.12 (1.95 – 2.21)
PN/NA	μm	0.81 ± 0.09 (0.65–0.91)	0.80 ± 0.08 (0.65 – 0.94)	0.81 ± 0.09- (0.67 – 0.87)
PN/PA	μm	0.45 ± 0.03 (0.39–0.49)	0.45 ± 0.03 (0.38 – 0.49)	0.45 ± 0.03 (0.40 – 0.47)

^*^AK = area of kinetoplast; AN = area of nucleus; AT = area of trypomastigote; BW = width of body through center of nucleus; FF = length of free flagellum; KN = kinetoplast to center of nucleus; NA = center of nucleus to anterior end; PA = total length without free flagellum; PK = posterior end to kinetoplast; PN = posterior end to center of nucleus; AN/AT = nuclear area index; BW/PA = body width index; PK/PA, PN/NA, PN/PA = nuclear index; PN/KN = kinetoplast index.

^**^Mean ± SD are provided, followed in parentheses by the minimum and maximum values.

Different superscript letters (a, b) within each morphometric feature indicate significant differences between morphotypes (*p* < 0.05).

**Table 3:** Continued.

Features*	Unit	Measurements**	
		SEBL-IV (*n* = 4)	SEBS (*n* = 4)
AK	μm^2^	1.01 ± 0.06 (0.94–1.06)	0.87 ± 0.18 (0.63–1.02)
AN	μm^2^	22.71 ± 6.86 (15.71–31.55)^ab^	17.70 ± 3.85 (13.14–22.55) ^b^
AT	μm^2^	238.32 ± 32.48 (200.08–275.93)^ab^	178.03 ± 55.53 (96.26–219.53) ^b^
BW	μm	7.12 ± 1.53 (5.90–9.37)^ab^	5.46 ± 0.17 (5.20–5.58) ^b^
FF	μm	4.77 ± 1.67 (2.93–6.86)	4.96 ± 0.59 (4.17–5.49)
KN	μm	14.92 ± 0.77 (14.01–15.71)	13.70 ± 1.33 (12.02–15.13)
NA	μm	37.73 ± 3.93 (34.45–43.19)	34.59 ± 2.17 (31.56–36.33)
PA	μm	66.73 ± 4.16 (62.81–70.94)	63.18 ± 5.54 (55.90–69.19)
PK	μm	14.95 ± 2.68 (12.73–18.49)	14.68 ± 1.99 (13.11–17.59)
PN	μm	29.42 ± 1.32 (28.23–31.28)	28.98 ± 2.92 (26.45–32.89)
AN/AT	μm	0.09 ± 0.02 (0.08–0.11)	0.10 ± 0.03 (0.08–0.14)
BW/PA	μm	0.11 ± 0.03 (0.09–0.15)	0.09 ± 0.01 (0.08–0.10)
PK/PA	μm	0.22 ± 0.04 (0.19–0.26)	0.23 ± 0.02 (0.21–0.25)
PN/KN	μm	1.98 ± 0.19 (1.80–2.23)	2.12 ± 0.18 (1.95–2.30)
PN/NA	μm	0.79 ± 0.09 (0.65–0.84)	0.84 ± 0.06 (0.79–0.91)
PN/PA	μm	0.44 ± 0.03 (0.41–0.47)	0.46 ± 0.02 (0.43–0.48)

^*^AK = area of kinetoplast; AN = area of nucleus; AT = area of trypomastigote; BW = width of body through center of nucleus; FF = length of free flagellum; KN = kinetoplast to center of nucleus; NA = center of nucleus to anterior end; PA = total length without free flagellum; PK = posterior end to kinetoplast; PN = posterior end to center of nucleus; AN/AT = nuclear area index; BW/PA = body width index; PK/PA, PN/NA, PN/PA = nuclear index; PN/KN = kinetoplast index.

^**^Mean ± SD are provided, followed in parentheses by the minimum and maximum values.

Different superscript letters (a, b) within each morphometric feature indicate significant differences between morphotypes (*p* < 0.05).

Trypomastigotes of morphotypes SEBL-III (Fig. 1C) and SEBL-IV (Fig. 1D) showed deep purple cytoplasm with longitudinal striations and scattered pale spots. The kinetoplast was small, round to oval, and stained reddish-purple. The undulating membrane was distinct and generally well pronounced. The oval, pinkish-purple nucleus was located near the center of the trypomastigote. Free flagella of morphotypes SEBL-III and SEBL-IV were short, with lengths of 4.07 ± 0.80 μm and 4.77 ± 1.67 μm, respectively. Additionally, the morphotype SEBL-IV had pale or colourless cytoplasm at both posterior and anterior ends.

Trypomastigotes of morphotype SEBS (Figs. 2A–2D) were small with the length of posterior end to anterior end (PA) of 63.18 ± 5.54 μm and body with through the center of nucleus (BW) of 5.46 ± 0.17 μm (Table 3). The cytoplasm was stained purple and contained longitudinal striations and scattered pale spots. The kinetoplast was small, round to oval, and stained reddish-purple. The undulating membrane was distinct and generally well pronounced. The oval, pinkish-purple nucleus was located near the center of the trypomastigote. The free flagellum was short, at 4.96 ± 0.59 μm in length.

### Morphological characteristics of trypanosomes in Yellow-vent bulbuls

**Figure 3:**
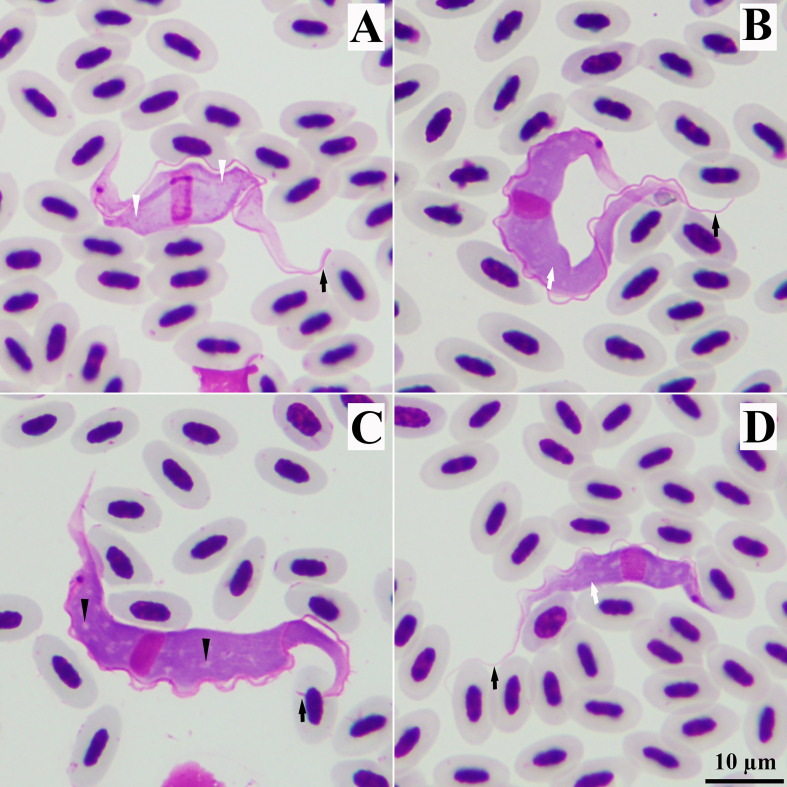
Large (A–C) and small (D) trypomastigote of *Trypanosoma* spp*.* found in Yellow-vent bulbuls (*Pycnonotus goiavier*). Large morphotypes of YVBL-I showed a pale purple cytoplasm with scattered azurophilic granules (A), YVBL-II showed a purple cytoplasm with scattered pale spots (B), YVBL-III showed a deep purple cytoplasm with scattered pale spots (C). Small morphotype YVBS showed a well pronounced undulating membrane (D). Note: Free flagellum = black arrow, longitudinal striations (myonemes) = white arrow, pale spots = black arrow head, azurophilic granules = white arrow head.

Trypanosomes found in SEBs showed four morphotypes (Table 2), including three large morphotypes (YVBL-I, YVBL-II, and YVBL-III, Figs. 3A–3C) and one small morphotype (SEBS, Fig. 3D). Trypomastigotes of morphotype YVBL-I showed pale purple cytoplasm containing longitudinal striations and scattered azurophilic granules (Fig. 2A). Additionally, the trypomastigote had a small, round to oval, reddish-purple kinetoplast and a centrally located oval pinkish-purple nucleus. Their undulation membrane was shallow. The free flagellum was short, at 4.09 μm (Table 4).

**Table 4:** Morphometry of four morphotypes of *Trypanosoma* sp. found in Yellow-vent bulbuls (*Pycnonotus goiavier*) from Thailand.

Features^*^	Unit	Measurements**			
		YVBL-I (n = 1)	YVBL-II (*n* = 3)	YVBL-III (*n* = 2)	YVBS (*n* = 4)
AK	μm^2^	0.89	1.09 ± 0.21 (0.86–1.28)	1.08 ± 0.11 (1.00–1.16)	0.99 ± 0.30 (0.76–1.43)
AN	μm^2^	22.83	21.43 ± 3.08 (19.32–24.97)	18.22 ± 0.61 (17.18–18.65)	16.24 ± 3.40 (11.32–18.96)
AT	μm^2^	181.95	249.71 ± 46.35 (217.02–302.76)	253.54 ± 48.92 (218.95–288.14)	132.77 ± 26.02 (99.33–162.60)
BW	μm	7.59	7.27 ± 1.34 (6.19–8.77)	6.65 ± 0.40 (6.36–6.93)	5.06 ± 1.05 (3.93–6.44)
FF	μm	4.09	5.61 ± 1.81 (3.80–7.42)	4.46 ± 1.77 (3.20–5.71)	9.77 ± 4.95 (6.02–16.92)
KN	μm	13.62	14.80 ± 1.11 (13.75–15.97)	14.78 ± 0.64 (14.33–15.24)	12.70 ± 1.76 (10.26–14.45)
NA	μm	23.09	35.68 ± 2.18 (33.76–38.05)	36.29 ± 0.36 (36.04–36.54)	27.07 ± 4.73 (20.36–31.46)
PA	μm	44.37	65.25 ± 1.39 (61.07–63.79)	65.59 ± 0.68 (65.10–66.07)	45.59 ± 8.13 (33.59–51.25)
PK	μm	8.07	14.25 ± 1.35 (12.98–15.66)	15.41 ± 0.46 (15.08–15.73)	6.72 ± 2.28 (3.35–8.29)
PN	μm	20.18	26.99 ± 1.45 (25.49–28.39)	30.38 ± 0.42 (30.08–30.68)	19.66 ± 3.55 (14.35–21.57)
AN/AT	μm	0.13	0.09 ± 0.01 (0.08–0.09)	0.07 ± 0.01 (0.06–0.08)	0.13 ± 0.03 (0.09–0.17)
BW/PA	μm	0.17	0.12 ± 0.02 (0.10–0.14)	0.10 ± 0.01 (0.10–0.010)	0.11 ± 0.03 (0.08–0.15)
PK/PA	μm	0.18	0.23 ± 0.03 (0.20–0.26)	0.23 ± 0.00 (0.23–0.24)	0.14 ± 0.03 (0.10–0.17)
PN/KN	μm	1.48	1.83 ± 0.12 (1.74–1.97)	2.06 ± 0.12 (1.97–2.14)	1.54 ± 0.12 (1.40–1.65)
PN/NA	μm	0.87	0.76 ± 0.08 (0.67 – 0.81)	0.84 ± 0.02 (0.82 – 0.85)	0.73 ± 0.04 (0.69–0.76)
PN/PA	μm	0.45	0.43 ± 0.03 (0.40 – 0.46)	0.46 ± 0.00 (0.46 – 0.46)	0.43 ± 0.01 (0.42–0.45)

*AK = area of kinetoplast; AN = area of nucleus; AT = area of trypomastigote; BW = width of body through center of nucleus; FF = length of free flagellum; KN = kinetoplast to center of nucleus; NA = center of nucleus to anterior end; PA = total length without free flagellum; PK = posterior end to kinetoplast; PN = posterior end to center of nucleus; AN/AT = nuclear area index; BW/PA = body width index; PK/PA, PN/NA, PN/PA = nuclear index; PN/KN = kinetoplast index.

**Mean ± SD are provided, followed in parentheses by the minimum and maximum values.

Trypomastigotes of morphotype YVBL-II showed purple cytoplasm containing longitudinal striations and scattered pale spots (Fig. 3B). The kinetoplast was small, round to oval, and stained reddish-purple. The oval, pinkish-purple nucleus was located centrally. Their undulation membrane was shallow. The free flagellum was short, at 5.61 ± 1.81 μm. Morphotype YVBL-III shared general characteristics with YVBL-II, but its cytoplasm was stained deep purple and the undulating membrane was well pronounced (Fig. 3C). The free flagellum was short, at 4.46 ± 1.77 μm.

Trypomastigotes of morphotype YVBS (Fig. 3D) were small, with the length of posterior end to anterior end (PA) of 45.59 ± 8.13 μm and body with through the center of nucleus (BW) of 5.06 ± 1.05 μm. The cytoplasm was stained purple and contained longitudinal striations and scattered pale spots. The kinetoplast was small, round to oval, and stained reddish-purple. The undulating membrane was distinct and generally well pronounced. The oval, pinkish-purple nucleus was located near the center of the trypomastigote. The free flagellum was 9.77 ± 4.95 μm in length.

### Comparative morphometry of trypanosomes in Thai bulbuls and other birds

Trypomastigotes of eight morphotypes found in Thai bulbuls had the length from posterior to anterior ends (PA) (Table 3 and 4) that fell within the range of PA reported from *Trypanosoma avium bakeri* (51.50–72.70 μm) (Table 5), except for morphotype YVBS, which had a smaller PA (33.59–51.25 μm). Additionally, no significant differences in PA were observed among trypanosome morphotypes in SEBs (*p* > 0.05). Overall, the PA lengths of these eight morphotypes exceeded those reported for *Trypanosoma avium*, *Trypanosoma pycnonoti*, and *Trypanosoma brimonti.*

**Table 5:** Morphometry of trypanosomes found in Pycnonotidae from previous reports.

Features^a^	Unit	Measurements				
		*T. avium bakeri* in *Pycnonotus jocosus* (*n* = 27)^b^	*T. avium* in Pycnonotidae (*n* = 9)^c^	*T. pycnonoti* found in *Pycnonotus tricolor* (*n* = 1)^d^	*T. brimonti* found in *Pycnonotus hainanus* (*n* = 1)^e^
AK	μm^2^	1.5 × 2.2^f^	–	1.00 μm^i^	–
AN	μm^2^	8.0 × 6.0^f^	12.8 ± 4.9^h^	–	–
AT	μm^2^	–	118.0 ± 20.1	–	–
BW	μm	7.00 (5.50–8.50)^g^	4.4 ± 1.2	5.40	4.70
FF	μm	16.50 (12.00–20.50)	–	–	6.60
KN	μm	–	10.5 ± 1.0	7.60	5.20
NA	μm	–	25.2 ± 2.6	24.30	14.50
PA	μm	61.50 (51.50–72.70)	47.0 ± 3.3	49.50	41.00
PK	μm	10.50 (6.50–12.50)	11.0 ± 2.7	12.20	11.50
PN	μm	25.00 (20.50–28.50)	21.8 ± 3.1	5.40	3.20
AN/AT	μm	–	0.10 ± 0.04	–	–
BW/PA	μm	–	0.10 ± 0.01	–	–
PK/PA	μm	–	0.23 ± 0.04	–	–
PN/KN	μm	–	0.21 ± 0.03	–	–
PN/NA	μm	–	0.87 ± 0.10	–	–
PN/PA	μm	–	0.46 ± 0.05	–	–

^a^AK = area of kinetoplast; AN = area of nucleus; AT = area of trypomastigote; BW = width of body through center of nucleus; FF = length of free flagellum; KN = kinetoplast to center of nucleus; NA = center of nucleus to anterior end; PA = total length without free flagellum; PK = posterior end to kinetoplast; PN = posterior end to center of nucleus; AN/AT = nuclear area index; BW/PA = body width index; PK/PA, PN/NA, PN/PA = nuclear index; PN/KN = kinetoplast index. ^b^Data reported by Chatterjee and Ray [14]. ^c^Data reported by Bennett, Earlé [4]. ^d^Data reported by Kerandel [25]. Note, number of trypomastigotes is not clear. ^e^ Data reported by Mathis and Leger [28]. ^f^Opposite diameters. ^g^Mean followed in parentheses by the minimum and maximum values. ^h^Mean ± SD. ^i^Diameter of kinetoplast.

The body width (BW) of morphotypes SEBS (5.46 ± 0.17 μm) was significantly different from that of morphotypes SEBL-I (7.96 ± 1.26 μm) and SEBL-II (7.58 ± 0.70 μm), *p* < 0.05. The area of trypomastigote (AT) of morphotypes SEBS (178.03 ± 55.53 μm^2^) was significantly different from that of morphotypes SEBL-I (271.75 ± 46.17 μm^2^) and SEBL-II (272.21 ± 32.24 μm^2^), *p* < 0.05. Additionally, the area of nucleus (AN) of morphotypes SEBS (17.70 ± 3.85 μm^2^) was significantly different from that of morphotypes SEBL-I (25.42 ± 3.15 μm^2^), *p* < 0.05.

The BW ranges of morphotypes SEBL-I (5.83–9.76 μm), SEBL-II (6.11–8.60 μm), SEBL-IV (5.90–9.37 μm), and YVBL-II (6.19–8.77 μm) were slightly greater than that reported for *T. a. bakeri* (5.50–8.50 μm). Additionally, the free flagellum of all trypanosome morphotypes detected in Thai bulbuls was shorter than that of *T. a. bakeri*.

### Molecular characteristics of trypanosomes in Thai bulbuls

**Figure 4:**
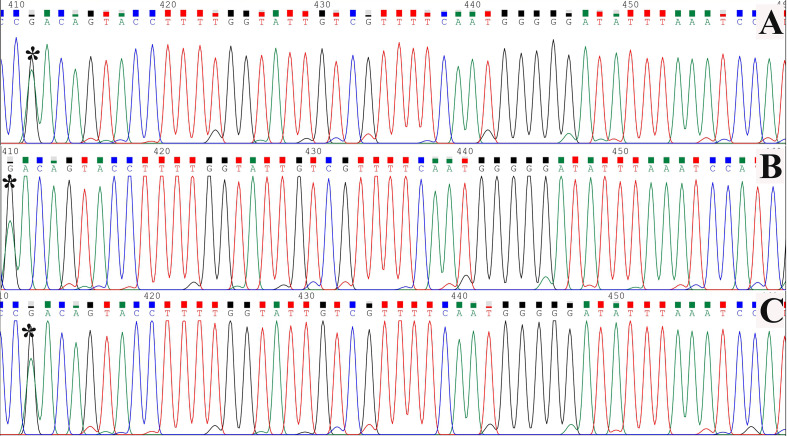
Electropherograms of small subunit ribosomal RNA (*SSU rRNA*) sequences of trypanosomes isolated from Streak-eared bulbuls (*Pycnonotus conradi*: WU23 (A), WU71 (B), and WU124 (C)), showing minimal double peaks (*).

Analysis of 21 sequences obtained from nested PCR amplification of the *SSU rRNA* gene revealed double or stutter peaks in all electropherograms, indicating co-infections with more trypanosome strains in all samples. Nevertheless, BLAST analyses confirmed that all 21 sequences corresponded to *Trypanosoma* sp. Due to the presence of co-infections, three sequences data with high-quality chromatograms, minimal double peaks, and no ambiguous bases (Fig. 4) were selected for further phylogenetic analysis. These selected sequences (*Trypanosoma* sp. WU23, WU71, and WU124) were considered as the dominant genotype among multiple genotypes detected in each mixed-infection sample.

**Figure 5:**
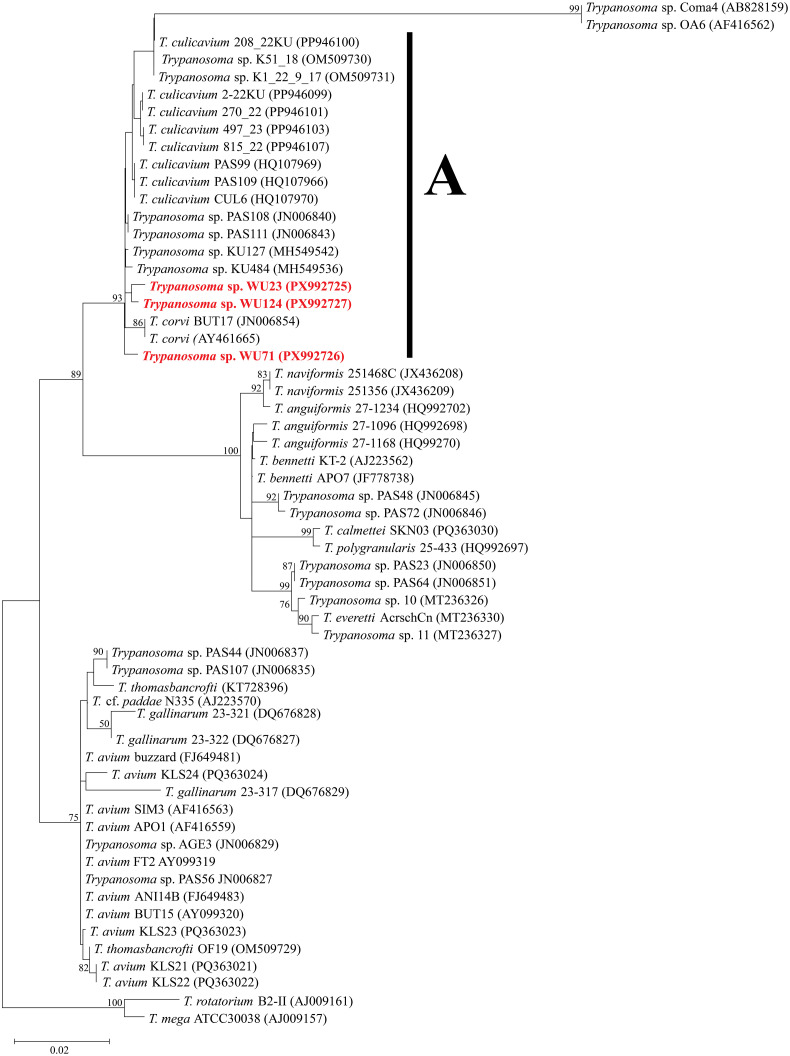
Maximum-likelihood phylogenetic analysis of partial sequences of the small subunit ribosomal RNA gene (*SSU rRNA*, 909 bp) of 57 avian trypanosomes, including our 3 sequences isolated from Streak-eared bulbuls (*Pycnonotus conradi*, red bold) and 54 sequences of avian trypanosomes from previous reports. Two amphibian trypanosomes (*Trypanosoma rotatorium and Trypanosoma mega*) were used as an outgroup. Posterior probabilities greater than 50% are shown at the phylogenetic node. A = *T. corvi/culicavium* clade.

Phylogenetic analysis (Fig. 5) revealed that *Trypanosoma* sp. WU23, WU71, and WU124 isolated from SEBs were grouped in the *Trypanosoma corvi*-*Trypanosoma culicavium* clade, with 88.50% to 100% homology. *Trypanosoma* sp. WU23 showed 97.26% similarity to *T. corvi* isolated from birds in Czechia (GenBank accession number: JN006854) and the United Kingdom (GenBank accession number: AY461665). Additionally, *Trypanosoma* sp. WU23 showed 97.39% to 99.71% similarity to *T*. *culicavium* isolated from *Culex* mosquitoes in Lithuania (GenBank accession number: PP946099–PP946101, PP946103, and PP946107) and Czechia (GenBank accession number: HQ107970), as well as birds in Czechia (GenBank accession number: HQ107966 and HQ107969).

Furthermore, *Trypanosoma* sp. WU71 and WU124 were closely related to *T. corvi* (JN006854 and AY461665), with similarity at 96.47% and 96.46%, respectively. *Trypanosoma* sp. WU71 was also closely related to *T*. *culicavium* (PP946099–PP946101, PP946103, PP946107, and HQ107969–HQ107970), with similarity ranging from 96.60% to 99.71%. Additionally, *Trypanosoma* sp. WU124 was closely related to *T*. *culicavium* (PP946099–PP946101, PP946103, PP946107, and HQ107969–HQ107970), with similarity ranging from 96.59% to 99.42%. Due to the presence of co-infections of different strains in all positive bulbuls tested, it was not possible to link the morphotypes with their genetic information.

Additionally, among avian trypanosomes isolated from vectors (*Culex* mosquitoes: PP946099–PP946101, PP946103, and PP946107; louse files: AF416562 and OM509729–OM509731; and biting midges: MT236326–MT236327), those from *Culex* mosquitoes and two louse files were phylogenetically grouped within the *T. corvi/culicavium* clade. The remaining trypanosomes from biting midges (MT236326–MT236327), and two from louse files (AF416562 and OM509729) were grouped within other clades.

## Discussion

In this study, a comprehensive morphological and molecular characterization of trypanosomes was developed. Molecular prevalence of trypanosomes in SEBs and YVBs in Southern Thailand was 11.39% and 75.00%, respectively, which is higher than the positive cases observed in microscopic examination (10.13% for SEBs and 62.50% for YVBs). This suggests that routine veterinary diagnostic laboratories should consider using higher sensitivity diagnostic tools, which could be PCR-based techniques or inexpensive buffy coat smears [12, 36]. The low number of trypomastigotes in blood smear was consistent with descriptions provided in previous studies [4, 31, 32], making it difficult to morphologically identify the parasites found and/or describe new species.

Furthermore, morphological description from the concentration method, such as buffy coat smear, was not recommended because morphological alterations can occur during centrifugation [12, 36]. Thus, identification of avian trypanosome species in point-of-care testing or veterinary diagnostic laboratories required alternative technologies, including rapid, user-friendly molecular assays and artificial intelligence-based whole-slide imaging. These approaches will become increasingly feasible as comprehensive morphological datasets are developed for machine-learning applications, as demonstrated for trypanosomes in other animal hosts [26]. Our data also suggest that infection in male and female bulbuls was not significantly different. However, further studies involving larger sample sizes is recommended.

This study provides the first report of morphological characteristics of trypanosomes infection in bulbuls from Thailand. However, it was not possible to identify the species of these trypanosomes. The SEBS morphotype (Fig. 2B) had trypomastigotes resembling *Trypanosoma avium bakeri* [14]. All SEBS morphotypes were found co-occurring with other morphotypes. Additionally, sequence analysis confirmed mixed infection, and the phylogenetic tree constructed using the dominant sequence grouped within the *T. corvi/culicavium* clade. The authors assume that this SEBS morphotype (Fig. 2B) may represent either multiple species infection or pleomorphic characteristics. Thus, further investigation was required.

Additionally, morphotype SEBL-IV (Fig. 1D) was similar to *Trypanosoma pycnonoti* [25], which was originally described from Dark-capped bulbul (*Pycnonotus tricolor*) in the Congo. Trypomastigotes of *T. pycnonoti* were fusiform and elongated, tapering at both ends, the anterior end being the more slender, and pale at the extremities [25]. The trypomastigotes of morphotype SEBL-IV found in this study showed pale posterior and anterior ends (Fig. 1D), but their morphometric features (Table 3) were greater than those of *T. pycnonoti* (Table 5). The authors assume that this variation might be due to the limited number of measured trypomastigotes, with only one specimen in the original description. Polymorphism of trypomastigotes was previously described in a report on *Trypanosoma corvi* [32]. It is recommended to conduct further studies to obtain additional evidence of polymorphism of trypanosomes infecting Thai bulbuls.

We found mixed-infections in all samples, together with several morphotypes in blood smears, suggesting high diversity of avian trypanosomes in the studied area. Notably, morphotypes SEBL-I and SEBL-II, which showed scattered distinct azurophilic granules (SEBL-I) and a shallow undulating membrane (SEBL-I and SEBL-II), may represent novel species. However, further investigation with additional samples and molecular characterization was required to confirm this. Although trypanosomes found in bulbuls can be classified into multiple morphotypes, they exhibit no major differences among these morphotypes, unlike trypanosomes in other vertebrates, such as anuran trypanosomes [61]. This made it challenging to distinguish between distinct species and pleomorphism in avian trypanosomes.

To solve the mixed-infection issue, DNA cloning might be needed to determine how many and which haplotypes of trypanosomes are present in these birds. In this study, DNA cloning was not conducted due to the limitation of time and cost. However, based on the assumption that conventional PCR and Sanger sequencing are capable of detecting a dominant genotype [38], some sequences (with high-quality chromatograms, minimal double peaks, and unambiguous bases (Fig. 4) were selected for phylogenetic analysis (Fig. 5). These dominant sequences phylogenetically clustered within the *Trypanosoma corvi-Trypanosoma culicavium* clade, suggesting the possible occurrence of undescribed trypanosomes in Thai bulbuls. Although more than 100 species of avian trypanosomes have been described, it is generally recognized that many of these taxa likely represent synonymous species and/or lack molecular characterization. Therefore, the trypanosome morphotypes observed in the present study should be interpreted with caution. The morphotypes exhibited only minor morphological differences, making it difficult to determine whether they represent distinct species or reflect the pleomorphic nature of avian trypanosomes. Additional molecular characterization, such as multilocus sequence analysis or whole-genome sequencing, combined with detailed morphological assessment, will be necessary to clarify their taxonomic status and determine whether the observed variation represents species-level diversity or intraspecific pleomorphism.

In Southern Thailand, *Culicoides huffi* and *Simulium chumpornense*, potential vectors of avian trypanosomes were found [37, 47, 55]. However, our phylogenetic analysis (Fig. 5) revealed that our samples grouped closely with trypanosomes isolated from *Culex* mosquitoes and louse files. Thus, future studies on vectors of avian trypanosomes should be conducted to confirm the vectors or discover other potential vectors found in the studied area. Furthermore, regular monitoring of trypanosomes in bulbuls and other commercially used passerine birds could help strengthen standard laboratory diagnostic protocols and competencies in wildlife and exotic pet services.

Walailak University has extensive green areas that provided suitable habitats, foraging grounds, and roosting sites for wild birds, with approximately 173 bird species recorded on campus [16]. As a result, further research on avian trypanosomes and other blood parasites on this campus may enhance our understanding and facilitate the discovery of additional parasite species, vectors, protozoan biology, and host-parasite interactions. In particular, the health impacts on bulbuls remain undocumented. Although this parasite was generally harmless, it could cause clinical symptoms [53] and pathological lesions [30] in some avian species.

## Conclusion

This study represents the first report of trypanosome infections in Streak-eared bulbuls (*Pycnonotus conradi*) and Yellow-vented bulbuls (*Pycnonotus goiavier*) in Thailand. The findings reveal the presence of multiple avian trypanosome morphotypes and demonstrate mixed infections involving multiple trypanosome strains and/or species, as confirmed by molecular analyses. Furthermore, the number of positive samples detected by molecular methods exceeded those identified through microscopic examination, highlighting the greater sensitivity of molecular diagnostics for detecting trypanosome infections. These findings contribute to the refinement of diagnostic protocols and strengthen laboratory capacity for the detection of hemoparasites in wildlife and exotic animal health services. Additional molecular characterization, including multilocus sequence analysis and whole-genome sequencing, combined with detailed morphological assessments, will be necessary to clarify the taxonomic status of the detected trypanosomes and determine whether the observed variation reflects species-level diversity or intraspecific pleomorphism. Nevertheless, the present study provides important baseline data for future investigations and contributes to a better understanding of parasite diversity, vector associations, protozoan biology, and host–parasite interactions in avian systems.
